# Association Between the Spheno-Occipital Synchondrosis and Mandibular Condyle Periphery Maturation in Relation to Chronological Age

**DOI:** 10.3390/dj14050298

**Published:** 2026-05-14

**Authors:** Zanda Bokvalde, Elizabete Domokejeva, Laura Neimane

**Affiliations:** 1 Department of Conservative Dentistry, Institute of Stomatology, Riga Stradins University, LV-1007 Riga, Latvia; 2 Department of General Dentistry, Riga Stradins University, LV-1007 Riga, Latvia; 3 Dental Clinic “Perle”, LV-1069 Riga, Latvia; 4 Department of Diagnostic Radiology, Institute of Stomatology, Riga Stradins University, LV-1007 Riga, Latvia

**Keywords:** mandibular condyle cortication (MCC), spheno-occipital synchondrosis (SOS), cone-beam computed tomography (CBCT), age assessment, craniofacial growth, forensic medicine

## Abstract

**Background:** Investigation of craniofacial growth and maturation, particularly of structures such as the spheno-occipital synchondrosis (SOS) and mandibular condyle cortication (MCC), provides valuable insight into late adolescent development. These markers may serve as valuable tools in age assessment, especially in legal and forensic contexts, where accurate determination of the 18-year threshold is critical. In addition, understanding their maturation can support more accurate assessment of skeletal development and improve clinical decision-making in growth-related dental treatments. **Methods:** This retrospective observational cross-sectional study was conducted to evaluate the stage of SOS and MCC maturation on a group of 230 individuals aged 14–22 years. Data was acquired from the mid-sagittal and sagittal sections of CBCT images representing, respectively, the SOS and the condyles. MCC was assessed bilaterally using a three-type system (Types I–III), and SOS fusion was evaluated using a four-stage system (Stages 0–3). Statistical analysis was performed to evaluate the association and correlation between the variables. **Results:** The Kruskal–Wallis test showed statistically significant differences in the age distribution of right and left MCC types, as well as SOS fusion stages (*p* < 0.001). Statistically significant differences were found in age distributions between all four SOS fusion stages in the MCC Type II groups of both condyles in both sexes, as well as Type III groups of the right condyle in both sexes and the left condyle in females (*p* < 0.001). Statistically significant differences were not observed in the MCC Type I group of the right and left condyle in both sexes and in the Type III group of the left condyle in males (*p* > 0.05). Spearman’s correlation analysis showed that the correlation between SOS fusion stages and MCC types was positive and statistically significant both between the parameters and with chronological age between sexes (r_s_ = 0.461–0.534, *p* < 0.001). **Conclusions:** This study revealed a statistically significant association and correlation between the maturation of the SOS, the MCC and chronological age. Simultaneous MCC Type III and SOS Stage 3 maturation was predominantly observed in individuals aged 18 years or older, although a small number of cases were identified below this threshold. SOS fusion and MCC may serve as skeletal parameters for age assessment; thus, they could be used as an adjunct method in a multifactorial age assessment procedure.

## 1. Introduction

Estimating the age of living individuals plays a crucial role in the field of forensic research, particularly in relation to criminal, civil and asylum proceedings. Forensic age assessment procedure is particularly important in criminal cases, since criminal responsibility and the legal age are defined differently across jurisdictions. In most countries, the legal age is 18, but this may differ depending on the region or country [1].

There is a growing need for reliable and accurate methods to identify both living individuals without age-verifying documents (such as a birth certificate or passport) in legal, forensic, and civil proceedings and cross-border migration cases, and deceased individuals in criminal cases and in anthropological research. Therefore, chronological age estimation has become of great importance in the identification of persons without a clear identity [1,2,3,4,5,6,7,8,9].

The chronological age of a person can be assessed using height, weight, and body mass index (BMI) data, signs of sexual maturation and mental development, pubertal status markers, and dental and skeletal maturation indicators [3,7,8,9,10]. Skeletal and dental maturation plays an essential role in chronological age assessment [3], while to assess skeletal age, ossification points are analyzed, including the maturation of the hand–wrist joint, medial clavicular epiphysis, and the joint surfaces of the pelvic bone. Recently, a few studies have examined the fusion of the cranial sutures [1,3,7,8], and this has potential as an additional forensic tool for age assessment [11].

Certain development sites, such as dentition and the hand–wrist joint, are known to assess the age below 14–15 years, but once their maturation is complete, it is difficult to assess age with the required accuracy [12]. Several studies have investigated third molar maturation as a potential marker for legal age assessment [13,14,15]. However, difficulties in assessing the age of an adolescent or young adult have highlighted the significance of investigating additional age assessment methods [12]. The complex influence of multiple endogenous and exogenous factors on the maturation of an individual tends to limit the use of currently available methods for precise age assessment. Despite the presence of various validated age assessment techniques, continued research into supplementary biological markers is essential to improve reliability, reduce uncertainty and strengthen multifactorial assessment protocols.

Cranial base synchondroses are important growth centers of the craniofacial skeleton [16,17,18]. The spheno-occipital synchondrosis (SOS) is considered a pivotal growth site due to its influence on the elongation of the basicranial axis, which provides a space for dento-alveolar development [19]. It exhibits relatively late ossification compared to other cranial base synchondroses that fuse prenatally (inter-sphenoid) or in early years of childhood (spheno-ethmoidal) [18,20]. The recent literature suggests that fusion of the SOS occurs between 17 and 20 years in both males and females across different populations [8,21,22,23]. Studies report that the closure of the SOS is partially affected by systemic hormonal changes that occur during puberty in a growing adolescent [24].

The temporomandibular joint (TMJ) is a synovial joint composed of the synovial cavity, the joint capsule, and the fibrocartilage articular disk, and is located between the mandibular fossa of the temporal bone and the mandibular condyle [25]. Maturation of mandibular condyles is closely related to the growth and development of the mandible [26,27,28], and it exhibits different morphological variations during an individual’s life [10,29]. During the maturation of mandibular condyles, a thick layer of fibrocartilage remains on the periphery of the joint surface, which thins by the age of 7 and transforms into a compact, continuous cortical bone layer in late teenage years as a result of the stress caused by dynamic mandibular movements. This process is referred to as mandibular condyle cortication (MCC) [25,30]. Previous studies conducted by Murali et al. (2024) and other authors have proven that the condyle cartilage is not displaced by bone texture until the attainment of adult skeletal maturity [7,10,11,26,27,28].

The recent literature includes studies on evaluating the SOS and the MCC in radiological examinations in the context of age assessment [8,11]. Correlating SOS maturation with MCC density is relevant, as both structures are influenced by skeletal maturation and growth-related remodeling, and their development occurs close to the legal age of 18 years. Understanding this relationship may help determine whether condylar bone characteristics can serve as an adjunctive indicator of skeletal maturity, potentially improving growth assessment.

## 2. Materials and Methods

### 2.1. Case Selection and Data Acquisition

This retrospective observational cross-sectional study was conducted to evaluate the stage of SOS and MCC maturation in 14–22-year-old individuals using CBCT images. The study was conducted in accordance with the Declaration of Helsinki and received approval by the Ethics Committee of Riga Stradins University (RSU) (2-PEK-4/535/2022).

CBCT scans were performed at the RSU Institute of Stomatology (RSU SI). The CBCT examinations included in this study were not performed for the purpose of this study. All scans were acquired based on different clinical indications, and the ALARA (As Low As Reasonably Achievable) principle was strictly followed in all cases. During the examination, a standardized positioning protocol was employed: individuals were seated upright with a neutral head position, focusing on a distant point at eye level, and positioned in the maximum intercuspation. The scans were performed as part of orthognathic surgery treatment purposes or for other clinical indications between 2018 and 2022. Patient data were received in a previously prepared Microsoft Office Excel file and securely stored and protected with password encryption to ensure compliance with data protection and confidentiality regulations.

The study included both male and female participants aged between 14 and 22 years (inclusive). Initially, CBCT images from 280 individuals (119 males and 161 females) were received and then selected based on the following inclusion and exclusion criteria (see Table 1).

**Table 1:** Inclusion and exclusion criteria for participant selection.

Inclusion Criteria	Individuals aged 14 to 22 years Radiographs clearly visualizing the SOS and mandibular condyles, without distortion or signs of abnormal morphology
Exclusion Criteria	Radiographs with inadequate sharpness or contrast or containing artifacts Radiographs incompletely visualizing the SOS and the mandibular condyles Pathological destructive changes on the surface of the mandibular condyles Suspected congenital or developmental craniofacial or skeletal deformities Suspected chronic or systemic diseases affecting jaw growth and development Other pathological conditions affecting the mandibular condyles

Information from patient medical records was not reviewed in this study.

After applying the inclusion and exclusion criteria, CBCT images from 230 of the 280 individuals were included in the study (135 females, 95 males).

### 2.2. Measurements and Data Analysis

The CBCT scans were performed using an i-CAT CBCT device (Imaging Sciences International, Hatfield, PA, USA). The standardized operating protocol was applied with the following parameters: 120 kVp, 5 mA, 17 × 9 cm–17 × 15 cm field of view (FOV), 0.3 and 0.4 vox. The obtained images were processed, reconstructed and analyzed using eXamVision 1.9.3.13 (KaVo Dental GmbH, Biberach, Germany). Radiological images were visualized using a 23.8-inch Dell monitor (model P2417Hc) with a resolution of 1920 × 1080 pixels. Data on the SOS fusion stages and the MCC types were collected and then statistically analyzed in a separate Microsoft Office Excel file.

**Figure 1:**
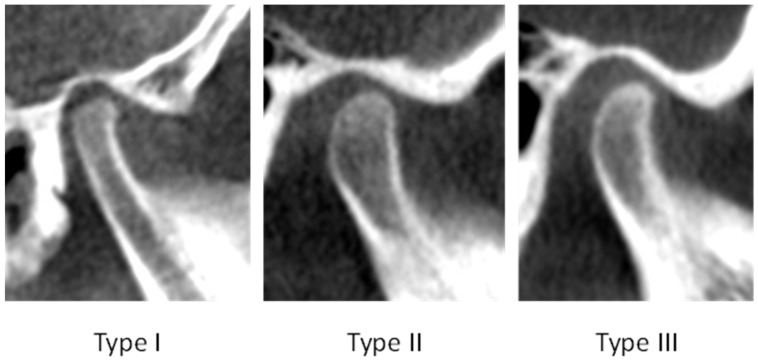
CBCT scans showing Type I, Type II and Type III cortication of the mandibular condyle.

The MCC type was determined in the sagittal plane of the CBCT image, where the condyles were observed completely and clearly, and their cortication was visualized at the highest clarity. The MCC was employed as an indicator of the periphery maturation of the mandibular condyles as it indicates the density differences between the cortical bone on the condylar surface and the surrounding bone structures. The MCC is classified according to a three-type system developed by Bayrak et al. (2018) (see Table 2 and Figure 1) [7].

**Figure 2:**
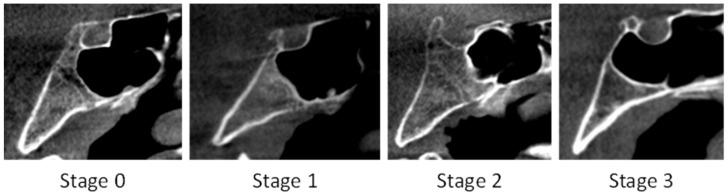
CBCT scans showing the SOS fusion stages (Stages 0–3).

The SOS fusion stage was assessed in the mid-sagittal plane of the image, and it was evaluated based on its fusion status. The extent of the SOS fusion was determined along a straight line crossing the center of the SOS, from the endocranial to the ectocranial side of the cranium, according to a four-stage system developed by Franklin et al. (2014) (see Table 3 and Figure 2) [20].

Image magnification was modified during the evaluation so that the operator could visualize the relevant structures completely and clearly. Images representing the SOS fusion stage and the MCC type were captured and saved in JPG format.

Prior to the evaluation, the primary operator underwent a calibration process involving a joint consensus review of 25 CBCT scans by all authors. Subsequently, the CBCT images were independently analyzed by one operator—a dentist with 9 years of clinical experience. In case of uncertainty, re-evaluation was performed together with other authors. Two weeks after the initial evaluation, re-evaluation of 25 randomly selected images was performed to verify intra-examiner reliability. During the evaluation, the chronological age and sex were not available to the operator in order not to influence the results of the study.

Each radiological image was analyzed for a maximum duration of 3 min. To prevent visual fatigue, no more than 10 images were examined consecutively without a break. The operator was allowed to adjust the brightness, contrast, and magnification settings of the images as needed.

### 2.3. Statistical Analysis

Statistical analysis of the data was performed using the IBM SPSS Statistics for Windows, version 29.0. A statistical significance level of *p* < 0.05 was adopted. The variables used in the study correspond to categorical or qualitative nominal (sex) and ordinal (SOS fusion stages and MCC types) data types, as well as quantitative data (chronological age). Initially, descriptive statistical analysis was performed, including calculation of mean, standard deviation, median, interquartile range, and minimum and maximum values. Subsequently, inferential statistics were performed using the following methods:

- To assess the normality of the distribution of quantitative data (chronological age), the Shapiro–Wilk test was performed.
- Since chronological age deviated from a normal distribution in all groups, the non-parametric Kruskal–Wallis test (independent-samples Kruskal–Wallis) (H) was employed to evaluate the relationship between SOS fusion stages, MCC types, and chronological age. In cases where the sample size was too small within specific SOS stages and MCC type groups, the Mann–Whitney U test (independent-samples Mann–Whitney U) (U) was performed.
- Spearman’s rank correlation coefficient (r_s_) was used to assess the correlation between SOS fusion stages, MCC types, and chronological age across sexes.
- Intra-examiner reliability was evaluated using the weighted Cohen’s Kappa coefficient (k).

## 3. Results

The study group included 230 individuals (135 females, 95 males) between the ages of 14 and 22. The mean age across both sexes was 17.63 ± 2.4 years, and it was 17.64 ± 2.5 years in the female group and 17.61 ± 2.3 in the male group. The age and sex distribution is shown in Table 4.

The weighted Cohen’s Kappa coefficient indicated a moderate level of intra-examiner reliability for the assessment of the SOS fusion stage, a very high level of intra-examiner reliability for the assessment of the right MCC type, and a moderate level of intra-examiner reliability for the assessment of the left MCC type (κ = 0.579, *p* < 0.001; κ = 0.844, *p* < 0.001; and κ = 0.507, *p* = 0.002, respectively).

The chronological age distributions across right and left MCC types according to sex are shown in Tables S1 and S2 in the Supplementary Materials. The mean age in the right MCC Type I group for males and females was the lowest at 14.67 ± 0.71 years and 14.00 ± 0.00 years, respectively, while in the left MCC Type I group, it was 14.71 ± 0.76 years and 14.33 ± 0.58 years, respectively. The mean age in the right MCC Type III group for males and females was the highest at 19.10 ± 1.90 years and 18.66 ± 2.21 years, respectively, while in the left ACL Type III group, it was 19.42 ± 1.79 years and 18.75 ± 2.22 years, respectively. It can be concluded that there are statistically significant differences between age distributions both among all right MCC types in the male (Kruskal–Wallis test, H = 36.98, *p* < 0.001) and female (Kruskal–Wallis test, H = 42.16, *p* < 0.001) groups, as well as among all left MCC types in male (Kruskal–Wallis test, H = 37.12, *p* < 0.001) and female (Kruskal–Wallis test, H = 33.89, *p* < 0.001) groups.

The chronological age distribution across SOS stages according to sex is shown in Table S3 in the Supplementary Materials. The mean age in the SOS Stage 0 group for males and females was 15.14 ± 1.28 years and 14.00 ± 0.00 years, respectively, while in the Stage 3 group, it was 19.42 ± 1.70 years for males and 18.86 ± 2.08 years for females. It can be concluded that there are statistically significant differences between age distributions across all SOS fusion stages in the male (Kruskal–Wallis test, H = 54.84, *p* < 0.001) and female (Kruskal–Wallis test, H = 60.80, *p* < 0.001) groups.

The chronological age distribution in relation to SOS fusion stages in the right and left MCC type groups according to sex is shown in the Supplementary Material Tables S4 and S5, respectively. There were no statistically significant differences between the SOS fusion stages in both right and left MCC Type I groups for both sexes, partially because of the too small sample size (*p* > 0.05). The mean age for females was smaller in almost all MCC type groups; it was also observed that the mean age is slightly higher in left than in right MCC type groups.

- The mean age in the right MCC Type II group increased from 14.92 ± 1.08 years at Stage 0 to 18.42 ± 1.73 years at Stage 3 for males, and from 14.00 ± 0.00 years at Stage 0 to 17.60 ± 2.06 years at Stage 3 for females. Statistically significant differences in age distributions were observed across SOS fusion stages in the right MCC Type II group, both for males (Kruskal–Wallis test, H = 24.28, *p* < 0.001) and females (Kruskal–Wallis test, H = 17.05, *p* < 0.001).
- The mean age in the right MCC Type III group increased from 17.33 ± 0.58 years at Stage 0 to 19.88 ± 1.51 years at Stage 3 for males; however, there were no females with Stage 0 SOS fusion in this group, so the mean age increased from 15.00 ± 0.00 years at Stage 1 to 19.13 ± 2.00 years at Stage 3 for females. Statistically significant differences in age distribution were observed across SOS fusion stages in the right MCC Type III group, both for males (Kruskal–Wallis test, H = 13.98, *p* = 0.003) and females (Kruskal–Wallis test, H = 19.58, *p* < 0.001).

Similar findings were observed in the left MCC type groups:

- The mean age in the left MCC Type II group increased from 15.07 ± 1.33 years at Stage 0 to 18.64 ± 1.82 years at Stage 3 for males; however, there were no females with Stage 0 SOS fusion in this group, so the mean age increased from 14.75 ± 0.58 years at Stage 1 to 18.15 ± 1.97 years at Stage 3 for females. Statistically significant differences in age distribution were observed across SOS fusion stages in the left MCC Type II group, both for males (Kruskal–Wallis test, H = 27.16, *p* < 0.001) and females (Kruskal–Wallis test, H = 28.25, *p* < 0.001).
- The mean age in left MCC Type III group increased from 17.00 ± 0.00 years at Stage 0 to 19.88 ± 1.48 years at Stage 3 for males; however, again, there were no females with Stage 0 SOS fusion in this group, so the mean age increased from 15.00 ± 0.00 years at Stage 1 to 19.17 ± 2.07 years at Stage 3 for females. No statistically significant differences in age distribution were observed across SOS fusion stages in the left MCC Type III group for males (Kruskal–Wallis test, H = 7.32, *p* = 0.062). This could be partially explained by the insufficient number of individuals in SOS fusion stage groups 0 and 1; however, statistically significant differences were observed for females (Kruskal–Wallis test, H = 12.59, *p* = 0.002).

Regarding the right and left MCC Type III groups for males, the small number of individuals with Stages 0 and 1 SOS fusion should be noted; individuals with Stage 0 had a higher mean age than individuals with Stage 1, which could indicate atypical maturation or a possible pathological condition in the individuals concerned. As mentioned, there were no females at all in the SOS fusion Stage 0 group in the MCC Type III group.

The correlation across SOS fusion stages, MCC types, chronological ages and sex is shown in Table 5. A moderate, positive and statistically significant correlation was observed across SOS fusion stages and right MCC types both in male and female groups (r_s_ (95) = 0.512 and r_s_ (135) = 0.508; *p* < 0.001, respectively), whereas a moderate, positive and statistically significant correlation was observed across SOS fusion stages and left MCC types in the male group (r_s_ (95) = 0.534, *p* < 0.001), but there was a low, positive and statistically significant correlation in the female group (r_s_ (135) = 0.461, *p* < 0.001). It can be observed that with increasing chronological age, the SOS fusion stage increases from Stage 0 to Stage 3, as does the MCC type from Type I to Type III. Spearman’s correlation coefficients (r_s_), representing effect size, ranged from 0.461 to 0.534, indicating low-to-moderate associations. This correlation is observed in both sexes.

## 4. Discussion

For living individuals without age-verifying documentation, age assessment is performed to estimate chronological age, particularly in forensic and legal contexts, including the management of cross-border migration cases [1,3,4,5,7,8]. The protocol for age assessment within the European Union is described in the “EASO Practical Guide on Age Assessment”. This process is characterized by a multidisciplinary approach, combining the examination of various non-medical and medical factors. The medical method, which involves application of radiation, includes radiological evaluation of the wrist, clavicle or collar bone and teeth development [31]. In this study, the maturation stage of two skeletal parameters—the SOS and the mandibular condyle periphery—was analyzed and their potential role and applicability in chronological age assessment was evaluated.

The findings of the present study indicate that the majority of individuals simultaneously presenting with fully matured skeletal structures–MCC Type III and SOS Stage 3, respectively—exceeded the legal age of adulthood in both male and female groups, with mean ages of 19.88 years in males and 19.13 years in females for the right condyle and 19.88 years in males and 19.17 years in females for the left condyle. Across all analyzed groups, females reached skeletal maturation earlier than males, with the mean age differences of approximately half a year.

The findings of current studies available in the scientific literature regarding SOS fusion in order to assess chronological age are presented in Table S6 in the Supplementary Materials. Similarly to this study, other studies have shown that complete maturation of the SOS occurs around the legal age, with females reaching maturity approximately two years earlier than males [1,4,11,20,32]. It has been reported that SOS fusion may occur later (Stage 2 at 25 years [5]) or earlier (Stage 3 at 13 years [5]; 13.42 years in males and 11.75 years in females [20]) than the average age for each stage. This is important to take into account, as the age of the youngest and oldest individuals within each SOS fusion stage group can influence the mean age values. Thus, differences in mean ages at each stage and the generally lower mean age in females compared to males may result from unequal sample distributions across populations [5]. Moreover, discrepancies in the reported age of SOS fusion across studies may also be explained by the use of different methodologies to analyze SOS fusion [24].

The findings of studies available in the scientific literature regarding the age at which MCC occurs are shown in Table S7 in the Supplementary Materials. Similarly to this study, other studies have shown that the MCC occurs around the legal age, with it being observed earlier in females than in males [7,8,11,26,33].

To date, the only studies available in the literature which consider the relationship between both skeletal parameters—the SOS fusion stage and the MCC type—in relation to chronological age are those by Bayrak et al. (2018) and Murali et al. (2024) [7,11]. In the former study, a positive correlation was observed between the SOS fusion stage and the MCC type. It should be noted that 76.6% of patients with Stage 0 SOS fusion exhibited Type I MCC. Among patients with Stage 3 fusion, 40.3% had Type II and 54.0% had Type III MCC. In the MCC Type I group, only eight patients exhibited Stage 3 SOS fusion [8]. Similar results were observed in this study—in both the right and left MCC Type I groups, 60% of individuals were at Stage 0, while in the MCC Type III group, the majority of individuals were at Stage 3 (75.8% in the right MCC Type III group and 79.2% in the left MCC Type III group). Murali et al. (2024) found in their study that there is a moderate to high positive statistically significant correlation between SOS fusion stages and both the right and left MCC types in both sexes [11]. When analyzing their results, the authors highlighted the minimum age of individuals, specifically noting that Type III MCC in females is observed from the age of 16, while in males it is observed from the age of 19. Similarly, it was observed that SOS fusion begins earlier in females than in males (Stage 1 in females is observed as early as 10 years of age, while in males it occurs only from 15 years of age). Furthermore, no individuals with Stage 1 SOS fusion were observed in the right MCC Type III group [11].

Similarly to other studies, this study also found that younger individuals exhibit lower stages of skeletal maturity, while older individuals show higher stages, indicating a positive correlation between the maturation of the studied skeletal parameters and chronological age. However, the authors observe that distributional differences alone do not demonstrate predictive utility and therefore interpret these findings as indicative of correlation between age and morphological stages rather than evidence of standalone age prediction. It should be noted that the age range of the individuals included in other studies was wider than that in this study, which explains the differences in the mean age for the lowest SOS fusion stages and the MCC type groups. The results of this study regarding the mean time of the SOS and the mandibular condyle periphery maturation exhibit the greatest similarity with findings from studies on Australian and Turkish populations [7,20]. The similarity with data suggests that SOS and MCC reflect a relatively conserved pattern of late craniofacial skeletal maturation. In addition, the use of comparable classification systems may contribute to consistent staging outcomes across different studies. Furthermore, as SOS and mandibular condyle periphery maturation occurs within a narrow late adolescent age window, inter-population biological variability is generally limited, which may further explain the observed agreement even across geographically distinct cohorts.

In this study, a slight bilateral asymmetry regarding the maturation of the mandibular condyle periphery was observed, specifically that the maturation of the right mandibular condyle periphery occurs earlier than that of the left. This could be explained by a physiologically normal deviation range or an error in the image acquisition or processing by the CBCT system [34]. However, this observation requires further investigation using a more substantial sample size to determine if these differences represent a consistent biological pattern.

Notably, it is important to address that even when MCC Type III and Stage 3 SOS are met simultaneously, an individual may still be younger than 18 years old (the youngest individual observed that exhibited Type III MCC and Stage 3 SOS fusion was 15 years old). In the present study, three individuals (one male and two females) exhibited MCC Type III bilaterally and Stage 3 SOS simultaneously while being under 18 years of age. This finding is recognized as a limitation and underscores the need for further studies to evaluate its consistency in a broader sample size. Furthermore, while comparisons of mean age per maturation stage provide useful contextual information, they should not be interpreted as precise age estimates for individual assessment.

The ambiguous results could suggest that multiple factors influence skeletal development. Skeletal maturation is affected by socioeconomic development, which is related to nutritional status and/or chronic diseases, which in turn are associated with disruptions in normal growth [5]. It is known that there are differences between populations in terms of ethnic, genetic, geographical, and environmental factors. Such variations may be related to factors such as vitamin D status influenced by sunlight exposure and latitude, climate-related lifestyle conditions, dietary intake including calcium and protein, and overall nutritional status during growth. Additionally, socioeconomic conditions, general health status, and the timing of pubertal development may further contribute to inter-population variability in skeletal maturation [3,7]. These factors are important when assessing skeletal maturation [8]. Therefore, the authors suggest that these methods should be applied with caution, and in accordance with EASO guidelines [31].

As mentioned in previous studies, SOS fusion stages may serve as an age-associated maturation indicator, and the positive correlation between SOS fusion stage and MCC type indicates that MCC may also be a useful method for age assessment [8,11]. Similarly to the findings of Bayrak et al. (2020) and Murali et al. (2024), the results obtained in this study indicate that as one skeletal parameter matures, other skeletal parameters also mature. Therefore, simultaneously evaluating SOS maturation stage and MCC in chronological age assessment could increase the reliability of the assessment [8,11].

This study has several limitations. To ensure that the results sufficiently represent the population, a larger sample size and wider age range are required. The limited number of observations in specific stage combinations, specifically MCC Type I with SOS Stage 2 or 3, and MCC Type III with SOS Stage 0 or 1 (see Tables S4 and S5), weakens the subgroup comparison and interpretation. Another limitation of the study was the uneven distribution of the age and sex. The age range of the studied sample is also of significant importance, as the mean age for SOS fusion stages and MCC types is partly dependent on the minimum and maximum age of the study sample. In particular, the mean age of the study population was close to 18 years, which may limit the precision of the results and the association strength between SOS, MCC maturation and chronological age. Especially relevant when compared with studies including a broader age range and, consequently, a wider distribution of mean ages at complete SOS fusion [8,24,32]. The authors acknowledge that, from a future research perspective, the implementation of multivariable analysis and formal diagnostic performance evaluation (e.g., sensitivity, specificity) would be valuable.

A subsequent limitation that should be considered is that the present method was evaluated in a specific study population and has not yet been externally tested. Therefore, its direct application to other populations or forensic contexts should be approached with caution. Additionally, the authors acknowledge that moderate levels of reproducibility may introduce potential limitations in the interpretations of SOS and MCC maturation stages, which may attenuate the strength of association and reduce interpretative precision, and therefore frame SOS and MCC maturation as complementary age assessment indications. In a forensic context, this moderate reproducibility implies that these markers should be used to establish an age range rather than a definitive chronological age to minimize the risk of age over- or under-estimation. Moreover, the authors recognize the limitation of CBCT imaging, as it cannot be ethically justified for routine age assessment in living individuals due to radiation exposure. However, this limitation does not apply in forensic contexts involving deceased individuals, where CBCT can be applied without radiation-related constraints. With respect to forensic applicability, the present findings suggest that SOS and MCC maturation stages provide biologically relevant age-associated information; however, their independent use for legal age determination is limited. The method is intended as an adjunctive tool in age assessment and should not be used as the primary basis for legal decision-making.

It is important to note the concerns about the representativeness of the study sample. The use of CBCT scans acquired for different clinical purposes may introduce some degree of selection bias, as the study population reflects a clinical rather than a population-based sample. Although the sample is not restricted to individuals with specific craniofacial anomalies, it may differ from the general population, potentially affecting the applicability of the findings.

The present study contributes to existing knowledge by providing population-specific data on SOS and MCC maturation stage and clarifying their correlation between morphological stages and chronological age. Assessment of SOS and MCC provides additional insight into late craniofacial maturation, particularly in the period approaching the end of skeletal growth. Their evaluation may improve clinical decision-making and treatment planning by assisting in determining whether craniofacial growth has been completed and better estimating residual growth potential, particularly in orthodontic and orthognathic treatment planning, as well as in cases where dental age estimation is insufficient to accurately assess skeletal maturation [24,35,36].

It would be beneficial to conduct a study such as this in other populations and compare the results to obtain a global perspective, taking all these factors into account. The findings of this study may serve as a foundation for the development of more extensive research in the future.

## 5. Conclusions

This study revealed a statistically significant association and correlation between the maturation of the SOS and mandibular condyle periphery and chronological age. As one skeletal parameter matures, the other skeletal parameter also matures; therefore, simultaneous analysis of SOS maturation and MCC in chronological age assessments may increase the reliability of the assessment.

Most individuals exhibiting full maturation of both skeletal structures simultaneously (MCC Type III and SOS Stage 3) were aged 18 years or older; however, a small number of cases below this threshold were observed. Maturation occurs earlier in females than in males.

SOS and MCC maturation should be regarded as supportive age-associated markers rather than standalone predictive tools for legal age determination.

## Figures and Tables

**Table 2:** Cortication type for assessment of mandibular condyle [7].

Type	Description
I	No cortication observed on the condyle
II	Bone on the superior surface of the condyle appears at a lower density than the structures around the condyle
III	The surface of the condyle appears at a higher or similar density than surrounding cortical areas

**Table 3:** Definition of features used to score the stage of spheno-occipital synchondrosis fusion [20].

Stage	Status	Description
0	Unfused	Completely open with no evidence of fusion between the basilar portion of the occipital and the sphenoid—no bone present in the gap
1	Fusing endocranially	No more than half the length of the synchondrosis is fused—proceeding endo- to ectocranially
2	Fusing ectocranially	Greater than half the length of the synchondrosis is fused—the ectocranial (inferior) border remains unfused
3	Complete fusion	Completely fused with the appearance of normal bone throughout—a fusion scar may be present

**Table 4:** Age and sex distribution.

Age (Years)	Males (n = 95)	Females (n = 135)	Total (n = 230)
14	10	16	26
15	9	22	31
16	15	13	28
17	15	14	29
18	11	13	24
19	11	25	36
20	13	12	25
21	6	7	13
22	5	13	18

**Table 5:** Correlation coefficients between SOS fusion stage, MCC type, and chronological age by sex.

	SOS Fusion Stage (r_s_)		Right MCC Type (r_s_)		Left MCC Type (r_s_)		Chronological Age (r_s_)	
	Male	Female	Male	Female	Male	Female	Male	Female
SOS fusion stage (r_s_)	1	1	0.512 **	0.508 **	0.534 **	0.461 **	0.759 **	0.673 **
Right MCC type (r_s_)			1	1	0.713 **	0.747 **	0.620 **	0.560 **
Left MCC type (r_s_)					1	1	0.626 **	0.499 **
Chronological age (r_s_)							1	1

** Correlation is statistically significant (*p* < 0.001).

## Data Availability

The original contributions presented in this study are included in the article and Supplementary Materials. Further inquiries can be directed to the corresponding author.

## Supplementary Materials

The following supporting information can be downloaded at: https://www.mdpi.com/article/10.3390/dj14050298/s1, Table S1: Descriptive values of chronological age according to the right MCC types by sex; Table S2: Descriptive values of chronological age according to the left MCC types by sex; Table S3: Descriptive values of chronological age according to the SOS fusion stage by sex; Table S4: Descriptive values of chronological age according to the SOS fusion stages in right MCC type groups by sex; Table S5: Descriptive values of chronological age according to the SOS fusion stages in left MCC groups by sex; Table S6: Summary of published scientific research results regarding the relationship between SOS maturation and chronological age; Table S7: Summary of published scientific research results regarding the relationship between MCC and chronological age.
