# Supplementary material for: Association Between the Spheno-Occipital Synchondrosis and Mandibular Condyle Periphery Maturation in Relation to Chronological Age
Source: Dent J (Basel). 2026 May 14;14(5):298. doi: 10.3390/dj14050298 (PMC13205414; doi:10.3390/dj14050298)
Supplement: Supplementary file 1 [file dentistry-14-00298-s001.zip › dentistry-4245932-supplementary.pdf]

Table S1. Descriptive values of chronological age according to the right MCC types by sex.

Sex	MCC type	Right condyle							*p*
		N	Mean	SD	Med	IQR	Min	Max	
Male	Type I	9	14.67	0.71	15.00	1 (Q1=14.00; Q3=15.00)	14	16	<0.001
	Type II	45	16.84	1.88	17.00	3 (Q1=15.50; Q3=18.00)	14	21	
	Type III	41	19.10	1.90	19.00	3 (Q1=17.50; Q3=20.50)	16	22	
Female	Type I	1	14.00	0.00	-	-	-	-	<0.001
	Type II	47	15.85	1.95	15.00	3 (Q1=14.00; Q3=17.00)	14	22	
	Type III	87	18.66	2.21	19.00	3 (Q1=17.00; Q3=20.00)	14	22	
Total	Type I	10	14.60	0.70	14.50	1 (Q1=14.00; Q3=15.00)	14	16	<0.001
	Type II	92	16.34	1.97	16.00	3 (Q1=15.00; Q3=18.00)	14	22	
	Type III	128	18.80	2.12	19.00	3 (Q1=17.00; Q3=20.00)	14	22	

Table S2. Descriptive values of chronological age according to the left MCC types by sex.

Sex	MCC type	Left condyle							*p*
		N	Mean	SD	Med	IQR	Min	Max	
Male	Type I	7	14.71	0.76	15.00	1 (Q1=14.00; Q3=15.00)	14	16	<0.001
	Type II	55	16.89	1.91	17.00	2 (Q1=16.00; Q3=18.00)	14	22	
	Type III	33	19.42	1.79	20.00	3 (Q1=18.00; Q3=21.00)	16	22	
Female	Type I	3	14.33	0.58	14.00	-	14	15	<0.001

	Type II	59	16.44	2.21	16.00	3 (Q1=15.00; Q3=18.00)	14	22	
	Type III	73	18.75	2.22	19.00	3 (Q1=17.00; Q3=20.00)	14	22	
**Total**	Type I	10	14.60	0.70	14.50	1 (Q1=14.00; Q3=15.00)	14	16	<0.001
	Type II	114	16.66	2.07	16.00	3 (Q1=15.00; Q3=18.00)	14	22	
	Type III	106	18.96	2.11	19.00	3 (Q1=17.00; Q3=20.25)	14	22	

Table S3. Descriptive values of chronological age according to SOS fusion stage by sex.

Sex	SOS fusion stage	*N*	Mean	SD	Med	IQR	Min	Max	*p*
**Male**	Stage 0	21	15.14	1.28	15.00	2 (Q1=14.00; Q3=16.00)	14	18	<0.001
	Stage 1	12	16.00	1.04	16.00	2 (Q1=15.25; Q3=16.75)	14	18	
	Stage 2	24	17.71	1.65	17.50	3 (Q1=16.25; Q3=18.75)	15	22	
	Stage 3	38	19.42	1.70	20.00	2 (Q1=18.75; Q3=21.00)	15	22	
**Female**	Stage 0	1	14.00	0.00	-	-	-	-	<0.001
	Stage 1	21	14.76	0.54	15.00	1 (Q1=14.00; Q3=15.00)	14	16	
	Stage 2	27	16.15	1.94	16.00	4 (Q1=14.00; Q3=18.00)	14	20	
	Stage 3	86	18.86	2.08	19.00	3 (Q1=17.00; Q3=20.00)	14	22	
**Total**	Stage 0	22	15.09	1.27	15.00	2 (Q1=14.00; Q3=16.00)	14	18	<0.001
	Stage 1	33	15.21	0.96	15.00	1 (Q1=15.00; Q3=16.00)	14	18	

	Stage 2	51	16.88	1.96	17.00	2 (Q1=16.00; Q3=18.00)	14	22	
	Stage 3	124	19.03	1.98	19.00	2 (Q1=18.00; Q3=20.00)	14	22	

Table S4. Descriptive values of chronological age according to the SOS fusion stages in right MCC type groups by sex.

**Right MCC type**	**SOS fusion stage**	**Male**							***p***
		N	**Mean**	**SD**	**Med**	**IQR**	**Min**	**Max**	
**Type I**	Stage 0	6	14.50	0.55	14.50	1 (Q1=14.00; Q3=15.00)	14	15	0.677
	Stage 1	2	15.00	1.41	15.00	-	14	16	
	Stage 2	1	15.00	0.00	-	-	-	-	
	Stage 3	0	-	-	-	-	-	-	
**Type II**	Stage 0	12	14.92	1.08	14.50	2 (Q1=14.00; Q3=16.00)	14	17	<0.001
	Stage 1	8	16.25	1.04	16.00	2 (Q1=15.25; Q3=17.00)	15	18	
	Stage 2	13	17.54	1.27	17.00	2 (Q1=16.50; Q3=18.50)	16	20	
	Stage 3	12	18.42	1.73	18.50	3 (Q1=17.00; Q3=20.00)	15	21	
**Type III**	Stage 0	3	17.33	0.58	17.00	-	17	18	0.003
	Stage 1	2	16.00	0.00	16.00	-	16	16	
	Stage 2	10	18.20	1.93	18.00	3 (Q1=16.75; Q3=20.00)	16	22	
	Stage 3	26	19.88	1.51	20.00	2 (Q1=19.00; Q3=21.00)	16	22	
**Right MCC type**	**SOS fusion stage**	**Female**							***p***
		N	Mean	SD	Med	IQR	Min	Max	
**Type I**	Stage 0	0	-	-	-	-	-	-	-
	Stage 1	1	14.00	0.00	-	-	-	-	

	Stage 2	0	-	-	-	-	-	-	
	Stage 3	0	-	-	-	-	-	-	
**Type II**	Stage 0	1	14.00	0.00	-	-	-	-	<0.001
	Stage 1	14	14.71	0.61	15.00	1 (Q1=14.00; Q3=15.00)	14	16	
	Stage 2	17	15.35	1.54	15.00	2 (Q1=14.00; Q3=16.00)	14	19	
	Stage 3	15	17.60	2.06	18.00	3 (Q1=16.00; Q3=19.00)	14	22	
**Type III**	Stage 0	0	-	-	-	-	-	-	<0.001
	Stage 1	6	15.00	0.00	15.00	0 (Q1=15.00; Q3=15.00)	15	15	
	Stage 2	10	17.50	1.84	17.50	3 (Q1=16.00; Q3=19.00)	14	20	
	Stage 3	71	19.13	2.00	19.00	3 (Q1=18.00; Q3=21.00)	15	22	

Table S5. Descriptive values of chronological age according to the SOS fusion stages in left MCC type groups by sex.

**Left MCC type**	**SOS fusion stage**	**Male**							***p***
		N	**Mean**	**SD**	**Med**	**IQR**	**Min**	**Max**	
**Type I**	Stage 0	5	14.60	0.55	15.00	1 (Q1=14.00; Q3=15.00)	14	15	0.192
	Stage 1	1	14.00	0.00	-	-	-	-	
	Stage 2	1	16.00	0.00	-	-	-	-	
	Stage 3	0	-	-	-	-	-	-	
**Type II**	Stage 0	14	15.07	1.33	14.50	2 (Q1=14.00; Q3=16.00)	14	18	<0.001
	Stage 1	10	16.20	0.92	16.00	1 (Q1=15.75; Q3=17.00)	15	18	
	Stage 2	17	17.35	1.27	17.00	2 (Q1=16.50; Q3=18.00)	15	20	

	Stage 3	14	18.64	1.82	19.00	3 (Q1=17.00; Q3=20.00)	15	22	
**Tips III**	Stage 0	2	17.00	0.00	17.00	-	17	17	0.062
	Stage 1	1	16.00	0.00	-	-	-	-	
	Stage 2	6	19.00	2.10	19.00	3 (Q1=17.50; Q3=20.50)	16	22	
	Stage 3	24	19.88	1.48	20.00	2 (Q1=19.00; Q3=21.00)	16	22	

**Left MCC type**	**SOS fusion stage**	**Female**							***p***
		N	**Mean**	**SD**	**Med**	**IQR**	**Min**	**Max**	
**Type I**	Stage 0	1	14.00	0.00	-	-	-	-	1.000 (Mann–Whitney)
	Stage 1	2	14.50	0.71	14.50	-	14	15	
	Stage 2	0	-	-	-	-	-	-	
	Stage 3	0	-	-	-	-	-	-	
**Type II**	Stage 0	0	-	-	-	-	-	-	<0.001
	Stage 1	16	14.75	0.58	15.00	1 (Q1=14.00; Q3=15.00)	14	16	
	Stage 2	17	15.41	1.58	15.00	3 (Q1=14.00; Q3=16.50)	14	19	
	Stage 3	26	18.15	1.97	18.00	2 (Q1=17.00; Q3=19.25)	14	22	
**Type III**	Stage 0	0	-	-	-	-	-	-	0.002
	Stage 1	3	15.00	0.00	15.00	-	15	15	
	Stage 2	10	17.40	1.90	17.50	3 (Q1=16.00; Q3=19.00)	14	20	
	Stage 3	60	19.17	2.07	19.00	3 (Q1=18.00; Q3=21.00)	15	22	

Table S6. Summary of published scientific research results regarding the relationship between SOS maturation and chronological age.

**Author**	**Year**	**Radiological examination method**	**Sex**	**Sample size**	**Age group**	**Country**	**Mean age of complete SOS fusion**

Bassed et al.[12]	2010	CT	Male, female	666	15-25	Australia	17.00^{c}
Can et al.[4]	2014	CT	Male, female	638	10-25	Turkey	17.00^{c}
Franklin et al.[20]	2014	MSCT	Male, female	312	5-25	Australia	19.83^{m}; 18.62^{f}
Lottering et al.[21]	2015	MSCT	Male, female	864	0-20	Australia	16.30^{m}; 13.80^{f}
Sinanoglu et al.[5]	2016	CBCT	Male, female	238	7-25	Turkey	20.00^{m}; 18.00^{f}
Ekizoglu et al.[1]	2016	MRI	Male, female	1078	7-21	Turkey	17.51^{c}
Demirturk Kocasarac et al.[22]	2016	CBCT, OPG	Male, female	349	8-25	Turkey	18.50^{m}; 20.56^{f}
Demirturk Kocasarac et al.[23]	2017	CBCT, lateral cephalometry, OPG	Male, female	116	8-28	Turkey	18.92^{m}; 18.02^{f}
Alhazmi et al.[24]	2017	CBCT	Male, female	741	6-20	United States	16.41^{m}; 15.25^{f}
Bayrak et al.[8]	2020	CBCT	Male, female	253	10-28	Turkey	16.38^{m}; 15.55^{f}
Sharma et al.[32]	2020	CBCT	Male, female	271	10-25	India	16.00^{c}
Murali et al.[11]	2024	CT	Male, female	435	10-25	India	22.39^{m}; 21.71^{f}

c—combined (both sexes); m—males; f—females

Table S7. Summary of published scientific research results regarding the relationship between MCC and chronological age.

Author	Year	Radiological examination method	Sex	Sample size	Age group	Country	Mean age in MCC Type I group	Mean age in MCC Type III group
Lei et al.[26]	2013	CBCT	Male, female	1438	10-30	China	$13\text{-}14^{m}$; $12\text{-}13^{f}$	22^{m}; 21^{f}

Bayrak et al.[7]	2018	CBCT	Male, female	433	8-31	Turkey	14.14^{m}; 13.06^{f}	19.39^{m}; 17.94^{f}
Bayrak et al.[8]	2020	CBCT	Male, female	253	10-28	Turkey	13.61^{m}; 12.34^{f}	17.67^{m}; 16.28^{f}
Seo et al.[33]	2022	CBCT	Male, female	829	13-25	Republic of Korea	$13\text{-}14^{c}$	24^{m}; 22^{f}
Murali et al.[11]	2024	CT	Male, female	435	10-25	India	12.84^{m}; 10.93^{f}	23.14^{m}; 22.69^{f}

c—combined (both sexes); m—males; f—females
